# A two-stage method for defect detection and mechanical damage assessment in mechanically harvested tobacco leaves

**DOI:** 10.3389/fpls.2026.1956974

**Published:** 2026-10-08

**Authors:** Erdeng Ma, Hezheng Wang, Siwei Zhang, Wanxin Li, Jiahui Liu, Dong Zhao, Jian Zhao

**Affiliations:** 1 Yunnan Academy of Tobacco Agricultural Sciences, Kunming, China; 2 School of Technology, Beijing Forestry University, Beijing, China; 3 Wenshan Branch of Yunnan Provincial Tobacco Company, Wenshan, Yunnan, China; 4 Key Lab of State Forestry Administration on Forestry Equipment and Automation, Beijing Forestry University, Beijing, China; 5 State Key Laboratory of Efficient Production of Forest Resources, Beijing, China

**Keywords:** computer vision, defect screening, harvest-related mechanical damage, mechanical harvesting, non-destructive assessment, tobacco leaf quality

## Abstract

Defects on mechanically harvested tobacco leaves may originate from diseases and pests or from harvesting operations, with distinct implications for crop quality. Dense small defects are easily missed, whereas local detection boxes cannot describe whole-leaf structural damage. We developed a two-stage vision method for non-destructive defect screening and assessment of harvest-related mechanical damage. Stage I used Tobacco Leaf Defect YOLO11n (TLD-YOLO11n), a task-adapted detector with high-resolution feature fusion, to identify four disease and pest categories and mechanical damage. Stage II used reference-midrib and leaf-morphology features to distinguish midrib breakage from lamina loss, while a relative lamina loss index provided an operational measure of tissue-loss severity. TLD-YOLO11n achieved mAP_0.5_ and mAP_0.5:0.95_ values of 84.5% and 64.1%, exceeding YOLO11n by 4.0 and 3.9 percentage points, respectively. Under controlled single-leaf imaging conditions, all 50 images with mechanical damage were passed to stage II, whereas none of the 156 disease- or pest-affected images were incorrectly passed. Damage type was correctly determined for 47 of the 50 mechanical-damage test images, yielding a joint accuracy of 94.0%. F1-scores for lamina loss and midrib breakage were 91.4% and 95.4%, respectively. The framework provides an approach for distinguishing harvest-related damage from other visual defects and quantifying loss of structural integrity in mechanically harvested tobacco leaves.

## 1 Introduction

Tobacco (*Nicotiana tabacum* L.) is an economically important crop, and its postharvest appearance and structural integrity directly affect leaf grading and downstream processing. Mechanically harvested leaves may retain disease lesions and pest-related symptoms acquired in the field and may also sustain mechanical damage, including lamina breakage, tissue tearing, and abnormal vein structures, during picking and transport (Chen et al., 2023; Tian et al., 2023; Sun et al., 2024a; Xiong et al., 2024). Disease symptoms and pest damage typically appear as local abnormalities in color, texture, and morphology, whereas mechanical damage is mainly characterized by lamina tissue loss or disrupted midrib continuity (He et al., 2023). These defect types differ markedly in both visual appearance and evaluation criteria. In evaluations of mechanical harvesting operations, leaf breakage rate is commonly considered together with missed-harvest rate and other indicators to assess equipment performance (Xin et al., 2023; You et al., 2026). However, simply distinguishing intact leaves from damaged leaves indicates only whether damage has occurred and does not identify its type or extent. Manual inspection is also susceptible to operator experience, fatigue, and imaging conditions, which can compromise the consistency of the evaluation results (Harjoko et al., 2019; Zhang et al., 2026; Zhao et al., 2026a). Therefore, a vision-based method is needed to detect diseases, pest damage, and mechanical damage in mechanically harvested tobacco leaves and to further determine the type and extent of mechanical damage.

Early visual analysis of tobacco leaves relied mainly on handcrafted color, shape, and texture features, combined with threshold segmentation, fuzzy comprehensive evaluation, or conventional classifiers for recognition and grading (Zhang and Zhang, 2011). With advances in deep learning, tobacco image analysis has gradually shifted from handcrafted-feature classification to deep feature learning. Lin et al. developed CAMFFNet for tobacco disease image recognition (Lin et al., 2022), whereas Wu et al. and Dai et al. used deep neural networks to assess tobacco leaf maturity (Dai et al., 2024; Wu et al., 2024). For tasks requiring both category identification and spatial localization, Sun et al. improved the You Only Look Once version 8 (YOLOv8) model for multiclass tobacco pest detection under complex backgrounds (Sun et al., 2024b); Zou et al. used semantic segmentation to improve the pixel-level recognition of small tobacco lesions (Zou et al., 2025); and Zhao et al. developed a lightweight model for the joint detection of tobacco diseases and damage (Zhao et al., 2026b). These studies demonstrate that deep learning can improve the automation of visual tasks involving tobacco diseases, pests, and maturity. However, these recent methods mainly focus on the recognition, localization, or segmentation of visual defects, whereas the present study further links multiclass defect detection with subsequent whole-leaf structural assessment of mechanically damaged leaves. Nevertheless, complex backgrounds, scale variation, dense small targets, and the loss of fine boundary information continue to limit detection performance, while accuracy must be balanced against computational complexity.

With the development of mechanized tobacco harvesting, machine vision has increasingly been applied to monitoring harvesting anomalies and quantifying leaf damage. You et al. combined an improved YOLOv10 model with digital-twin technology to identify missed tobacco plants, dropped leaves, and damaged stalks (You et al., 2026). Yu et al. used an improved YOLOv5 model to distinguish intact leaves from damaged leaves and aggregated multiframe detections using ByteTrack to estimate the overall damage rate of fresh tobacco leaves online (Yu et al., 2026). These studies demonstrate the potential of object detection for monitoring the quality of mechanized tobacco harvesting operations. However, their evaluations have focused mainly on harvesting anomaly categories, binary discrimination between intact and damaged leaves, or overall damage rates, without further characterizing specific damage forms in individual leaves. The bounding-box outputs of conventional object detectors provide category and localization information but do not directly encode whole-leaf structural descriptors such as midrib continuity or the proportion of lamina tissue loss. To quantify leaf tissue loss, Wang et al. combined image processing with deep learning to estimate herbivory-induced leaf damage, providing a basis for the automated assessment of leaf area loss (Wang et al., 2024b). However, such studies primarily address herbivory or general tissue loss, whereas mechanically harvested tobacco leaves may also exhibit structural damage, such as disrupted midrib continuity. Midrib breakage and lamina loss represent damage to the vascular support structure and loss of leaf tissue, respectively, and their structural differences cannot be fully captured by local bounding boxes alone. These tasks therefore involve different spatial information requirements: local feature detection is required to screen heterogeneous visual defects, whereas discrimination and quantification of mechanical damage depend on whole-leaf structural information. Accordingly, the present study adopts a task-decomposed two-stage strategy in which Stage I performs local multiclass defect detection and Stage II is conditionally activated for whole-leaf structural analysis, rather than using a single detection model to infer both local defect categories and global structural-damage descriptors. This design preserves the whole-leaf information required for structural assessment while restricting the secondary analysis to images identified as mechanically damaged.

To address these limitations, we propose a two-stage method for defect detection and mechanical damage assessment in mechanically harvested tobacco leaves. Stage I uses Tobacco Leaf Defect YOLO11n (TLD-YOLO11n) to detect five target classes: tobacco frogeye leaf spot, tobacco cucumber mosaic disease, oriental tobacco budworm, tobacco wildfire disease, and mechanical damage. When mechanical damage is detected, the corresponding whole-leaf image is passed to stage II. Stage II distinguishes midrib breakage from lamina loss using reference-midrib and leaf-morphology features and then provides a relative estimate of lamina loss for the operational classification of lamina-loss severity. The main contributions of this study are as follows:

1. A TLD-YOLO11n detector was developed by adding a P2 detection head and introducing Content-Aware ReAssembly of FEatures (CARAFE) upsampling to improve the preservation and fusion of high-resolution features. A dilation-wise residual (DWR) structure was embedded into C3k2 to form the C3k2_DWR module, which was further combined with the Simple, Parameter-Free Attention Module (SimAM) to enhance the representation of tobacco leaf defects with varying scales, textures, and morphologies.
2. The effectiveness of the proposed architecture was systematically evaluated through module ablation, comparisons among alternative modules, comparisons with other YOLO variants, and additional statistical validation, with emphasis on module effectiveness, model stability, and accuracy–complexity trade-offs.
3. A task-decomposed two-stage framework was established to connect local multiclass defect detection with whole-leaf structural damage assessment. Mechanical damage detected in Stage I conditionally triggered stage II, in which reference-midrib and leaf-morphology features were used to distinguish midrib breakage from lamina loss, and a relative lamina loss index was introduced to characterize the extent of tissue loss. Stage I mechanical damage screening was evaluated on the complete test set, and joint damage-type classification was assessed on the mechanical damage subset.

## 2 Materials and methods

### 2.1 Dataset construction and splitting

A tobacco leaf object-detection dataset containing disease symptoms, pest targets, and mechanical damage was constructed using publicly available and self-collected images. Disease and pest images were obtained from the Tobacco22 dataset available on Roboflow Universe (https://universe.roboflow.com/tobacco-kubzs/tobacco22; accessed 4 April 2026). The dataset contained four target classes: tobacco frogeye leaf spot, tobacco cucumber mosaic disease, oriental tobacco budworm, and tobacco wildfire disease. To standardize the class names, the original Pinyin labels were mapped to their corresponding English names, while the class identifiers and bounding-box annotations were retained. A total of 200 mechanical damage images were collected in Qujing, Yunnan Province, China using the built-in camera of a Huawei P50 smartphone. Images were acquired under ambient indoor illumination. During image acquisition, individual tobacco leaves were placed approximately flat on a black background, with the petiole oriented downward and the leaf tip upward. The public disease and pest images and the self-collected mechanical damage images were split at the original-image level into training, validation, and test sets at a ratio of 7:2:1. Within each subset, the mechanical damage images were augmented using random horizontal flipping, brightness adjustment, contrast adjustment, and their combinations. Each original image and all of its augmented versions were retained in the same subset. After augmentation, the final dataset comprised 2060 images, including 1560 disease- and pest-affected images and 500 mechanical-damage images. The dataset did not include a separate healthy or defect-free class; the 1560 disease- and pest-affected images represented non-mechanical defects for mechanical-damage screening. The final dataset contained 1442 training images, 412 validation images, and 206 test images, with 10247 annotated instances. The detector performance reported in **Tables 1**–**5** was evaluated on the 206-image test set. The image-level and annotated-instance distributions across the dataset splits are presented in **Table 6**, and representative samples are shown in **Figure 1**.

**Table 1:** Ablation results for the proposed optimization modules.

P2	CARAFE	C3k2_DWR	SimAM	P (%)	R (%)	mAP_0.5_ (%)	mAP_0.5:0.95_ (%)	Params (M)	GFLOPs	Model size (MB)
–	–	–	–	83.5	73.7	80.5	60.2	2.591	6.4	5.3
✓	–	–	–	84.1	75.0	82.3	61.3	2.667	10.4	5.6
–	✓	–	–	80.3	75.1	78.9	59.4	2.725	6.9	5.5
–	–	✓	–	82.1	75.6	82.7	61.9	2.624	6.6	5.3
–	–	–	✓	80.7	77.4	80.6	60.8	2.591	6.4	5.3
✓	✓	–	–	84.8	72.2	81.2	61.2	2.812	11.2	5.9
✓	✓	✓	–	82.7	76.0	82.4	61.5	2.847	11.5	5.9
✓	✓	✓	✓	87.0	77.8	84.5	64.1	2.847	11.5	5.9

✓Indicates that the module was included, whereas - indicates that it was excluded.

**Table 2:** Comparison of different upsampling methods.

Upsampling method	P (%)	R (%)	mAP_0.5_ (%)	mAP_0.5:0.95_ (%)	Params (M)	GFLOPs	Model size (MB)
Nearest-neighbor	83.6	75.2	81.3	61.3	2.703	10.7	5.7
DySample	87.0	75.3	83.4	62.5	2.717	10.7	5.7
CARAFE	87.0	77.8	84.5	64.1	2.847	11.5	5.9

**Table 3:** Detection performance of different C3k2 variants.

C3k2 module	P (%)	R (%)	mAP_0.5_ (%)	mAP_0.5:0.95_ (%)	Params (M)	GFLOPs	Model size (MB)
Original C3k2	85.0	74.3	82.1	60.6	2.812	11.2	5.9
C3k2_FDConv	82.1	79.6	83.3	61.5	2.871	11.3	7.8
C3k2_RepViTBlock	84.8	73.5	81.0	60.9	2.763	10.9	5.8
C3k2_FourierSR	85.5	72.7	81.8	59.5	2.594	10.5	5.4
C3k2_DWR	87.0	77.8	84.5	64.1	2.847	11.5	5.9

**Table 4:** Detection performance with different attention mechanisms.

Attention mechanism	P(%)	R(%)	mAP_0.5_ (%)	mAP_0.5:0.95_ (%)	Params (M)	GFLOPs	Model size(MB)	Inference time (ms/image)
None	82.7	76.0	82.4	61.5	2.847	11.5	5.9	6.0
CBAM	83.6	76.2	82.8	62.3	2.848	11.5	6.0	6.7
NAM	81.7	77.1	83.3	62.0	2.847	11.5	6.0	6.2
EMA	86.5	75.2	84.1	62.8	2.848	11.6	6.0	6.5
SimAM	87.0	77.8	84.5	64.1	2.847	11.5	5.9	6.3

**Table 5:** Detection performance of different YOLO models.

Model	P(%)	R(%)	mAP_0.5_ (%)	mAP_0.5:0.95_ (%)	Params (M)	GFLOPs	Model size (MB)	Inference time (ms/image)
YOLOv5n	82.1	74.7	80.9	58.9	2.509	7.2	5.1	4.4
YOLOv6n	81.4	73.6	79.7	60.1	4.239	11.9	8.3	3.5
YOLOv8n	84.5	73.2	80.3	60.0	3.012	8.2	6.0	3.8
YOLOv9t	79.3	76.5	81.0	61.0	2.006	7.9	4.5	11.1
YOLOv10n	79.1	74.0	78.7	58.7	2.709	8.4	5.5	4.7
YOLO11n	83.5	73.7	80.5	60.2	2.591	6.4	5.3	5.3
YOLOv12n	79.5	75.2	79.3	59.1	2.569	6.5	5.3	8.1
YOLOv26n	84.5	74.5	80.5	58.6	2.506	5.8	5.2	6.2
TGL-YOLO (n)	81.1	74.2	78.4	59.0	2.618	5.8	5.4	8.9
TLD-YOLO11n	87.0	77.8	84.5	64.1	2.847	11.5	5.9	6.3

**Table 6:** Distribution of images and annotated instances across the training, validation, and test sets.

Data level	Category	Train	Validation	Test	Total
Images	Disease- and pest-affected images	1092	312	156	1560
	Mechanical damage (midrib breakage)	231	66	33	330
	Mechanical damage (lamina loss)	119	34	17	170
	Total images	1442	412	206	2060
Annotated instances	Tobacco frogeye leaf spot	631	189	99	919
	Tobacco cucumber mosaic disease	1353	383	188	1924
	Oriental tobacco budworm	4355	1077	435	5867
	Tobacco wildfire disease	586	135	103	824
	Mechanical damage	511	139	63	713
	Total instances	7436	1923	888	10247

Midrib breakage and lamina loss are the two Stage II subcategories of mechanical damage; together, they comprise 500 mechanical-damage images. No separate healthy or defect-free image class was included. The 1560 disease- and pest-affected images represent non-mechanical defects in the mechanical-damage screening task.

**Figure 1:**
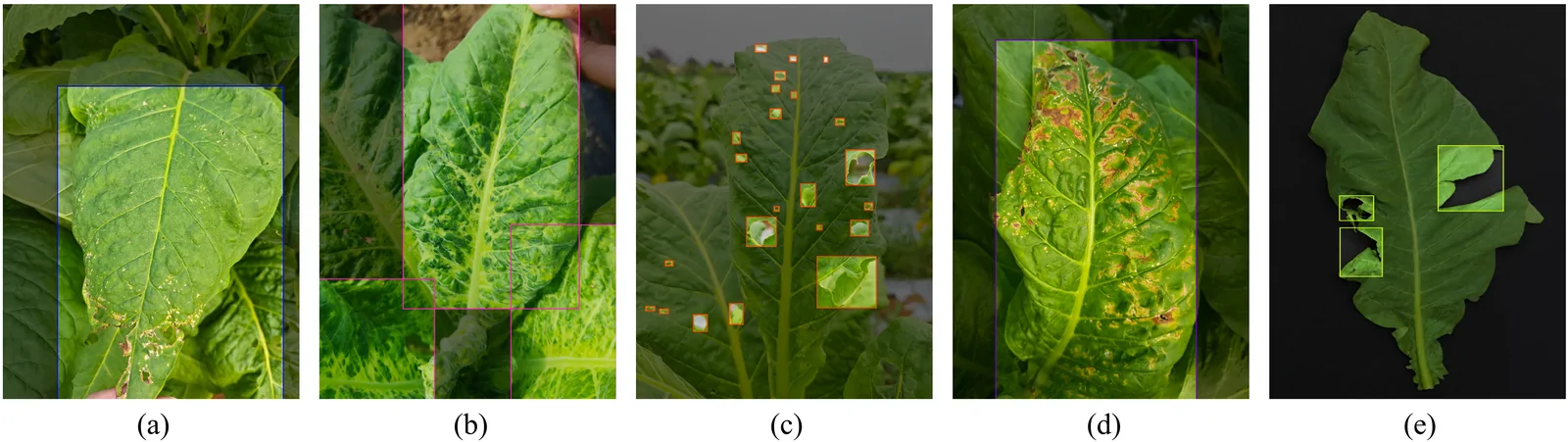
Representative tobacco leaf samples: **(a)** tobacco frogeye leaf spot; **(b)** tobacco cucumber mosaic disease; **(c)** oriental tobacco budworm; **(d)** tobacco wildfire disease; and **(e)** mechanical damage.

For Stage I, all mechanically damaged regions were uniformly annotated as mechanical damage. For Stage II, the corresponding mechanical damage images in the same dataset splits were additionally assigned image-level labels of either midrib breakage or lamina loss. After augmentation, the mechanical damage subsets contained 350 training images, 100 validation images, and 50 test images. The numbers of midrib breakage images were 231, 66, and 33 in the training, validation, and test subsets, respectively, whereas the corresponding numbers of lamina loss images were 119, 34, and 17. Thus, the 500 mechanical-damage images comprised 330 midrib breakage images and 170 lamina loss images. Mechanical-damage severity was further expressed using two operational categories, mild and severe. These severity categories were not independent image-level training labels: midrib breakage was operationally categorized as severe mechanical damage, whereas the severity of lamina loss was determined from the relative lamina loss index using the predefined 10% threshold described in Section 2.3.2. The two-stage cascade was evaluated on the full set of 206 test images. The 50 mechanical-damage images were used to evaluate mechanical-damage screening and stage II damage-type classification, whereas the remaining 156 images affected by diseases or pests were used to determine whether images with non-mechanical defects were incorrectly forwarded to stage II. For stage II, the 50 mechanical-damage test images were not used for feature standardization, classifier fitting, or decision-threshold selection.

### 2.2 Improved YOLO11n network

#### 2.2.1 Overall architecture of TLD-YOLO11n

YOLO11n is a single-stage object detector consisting primarily of a backbone, a neck, and detection heads. The backbone extracts hierarchical features using convolutional layers and C3k2 modules, while the Spatial Pyramid Pooling–Fast (SPPF) and C2PSA (C2 with Position-Sensitive Attention) modules provide contextual modeling. The neck fuses features at different scales, and the detection heads perform object classification and bounding-box regression. Tobacco diseases, pest damage, and mechanical damage differ substantially in scale, morphology, and texture. Early lesions, small insect-feeding holes, and local tears occupy relatively few pixels and are easily affected by complex backgrounds, leaf occlusion, illumination variation, and vein textures. Consequently, the original YOLO11n has limitations in extracting and fusing fine-grained multiscale features for these targets.

Based on these characteristics, TLD-YOLO11n (Tobacco Leaf Defect YOLO11n) was developed. DWR was embedded into the feature-transformation branch of C3k2 to form the C3k2_DWR module, which was deployed in the high-resolution P2 branch and at the bottom-up P3, P4, and P5 fusion nodes. SimAM attention was introduced after feature fusion at P2 (stride 4) and P3 (stride 8). CARAFE replaced nearest-neighbor upsampling in the top-down pathway of the neck. In addition, the P2 feature from the backbone was introduced into the neck, and a P2 detection head was added (Wang et al., 2025). The improved model performs predictions at four scales, namely P2, P3, P4, and P5. For an input size of 640 × 640 pixels, the P2 feature map has a spatial resolution of 160 × 160, which helps preserve detailed information from small lesions, insect-feeding holes, and local mechanical damage. The overall architecture is shown in **Figure 2**.

**Figure 2:**
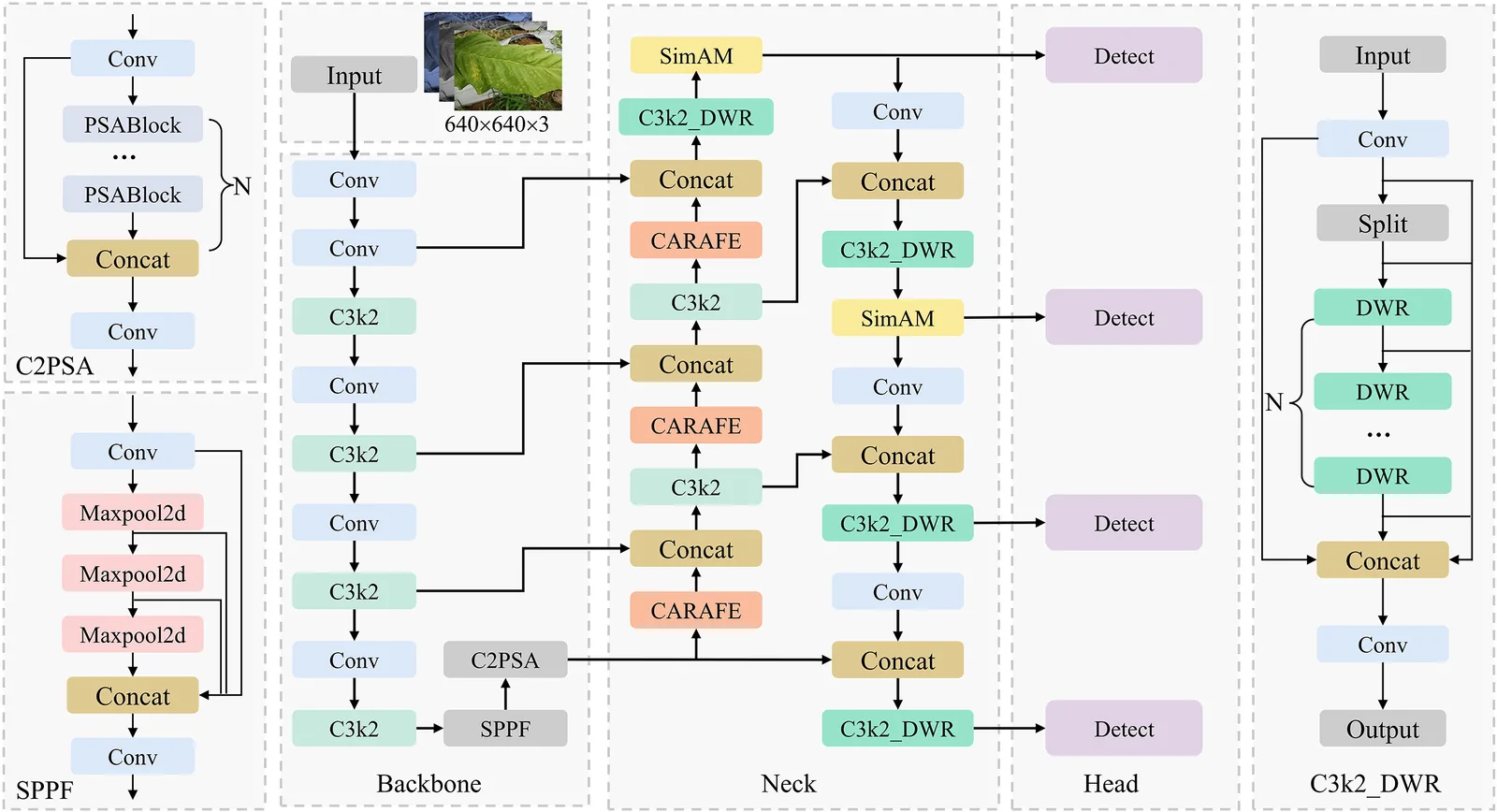
Overall architecture of TLD-YOLO11n.

#### 2.2.2 CARAFE upsampling

YOLO11n uses nearest-neighbor interpolation for upsampling in its neck. Although computationally efficient, this method copies features according to fixed spatial positions and cannot adapt the reassembly weights to local image content, potentially resulting in the loss of details along lesion boundaries, pest-related textures, and mechanical damage contours. In this study, CARAFE was used to replace the upsampling modules in the top-down pathway of the neck (Wang et al., 2019), as illustrated in **Figure 3**. CARAFE consists of a kernel-prediction module and a content-aware reassembly module. The kernel-prediction module first compresses the input channels using a 1 × 1 convolution and then extracts local contextual information through a content encoder. A reassembly kernel is predicted for each output position, followed by spatial rearrangement and Softmax normalization to obtain the corresponding weights. The content-aware reassembly module generates the upsampled feature map by computing a weighted sum over the local neighborhood of the input feature map. Unlike fixed interpolation, CARAFE adaptively reassembles features according to their local content, thereby preserving local structural information during multiscale feature fusion.

**Figure 3:**
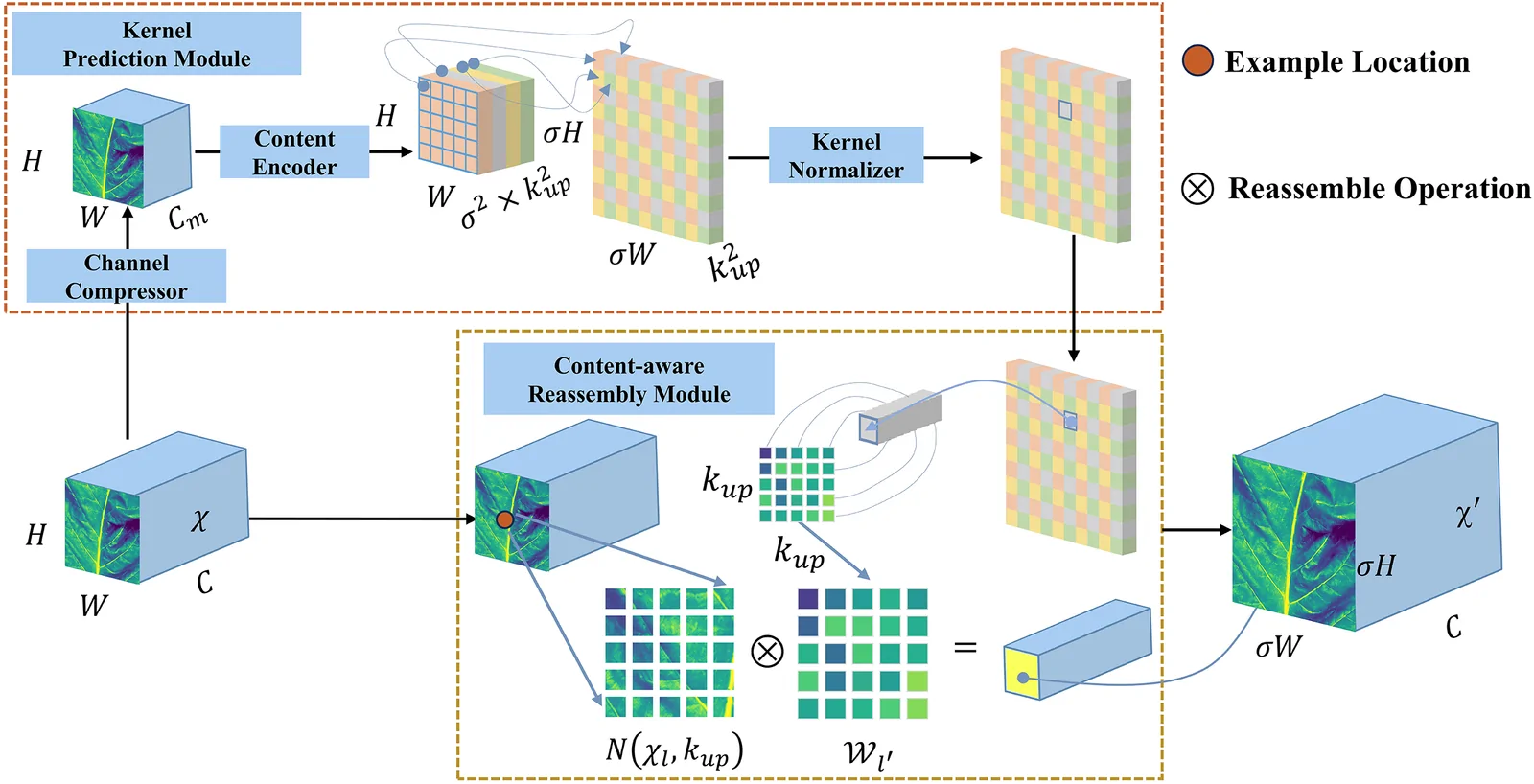
Structure of the CARAFE upsampling module.

#### 2.2.3 C3k2_DWR feature extraction module

Tobacco lesions, pest-affected regions, and mechanical damage targets differ in scale, morphology, and texture. Small lesions and insect-feeding holes are primarily characterized by local color or texture variations, whereas tears, holes, and marginal tissue loss usually exhibit irregular boundaries. To capture both local details and contextual information over different receptive fields, the multi-dilation residual structure of DWR was embedded into the feature-transformation branch of C3k2 to construct the C3k2_DWR module (Wei et al., 2022), as shown in **Figure 4**.

**Figure 4:**
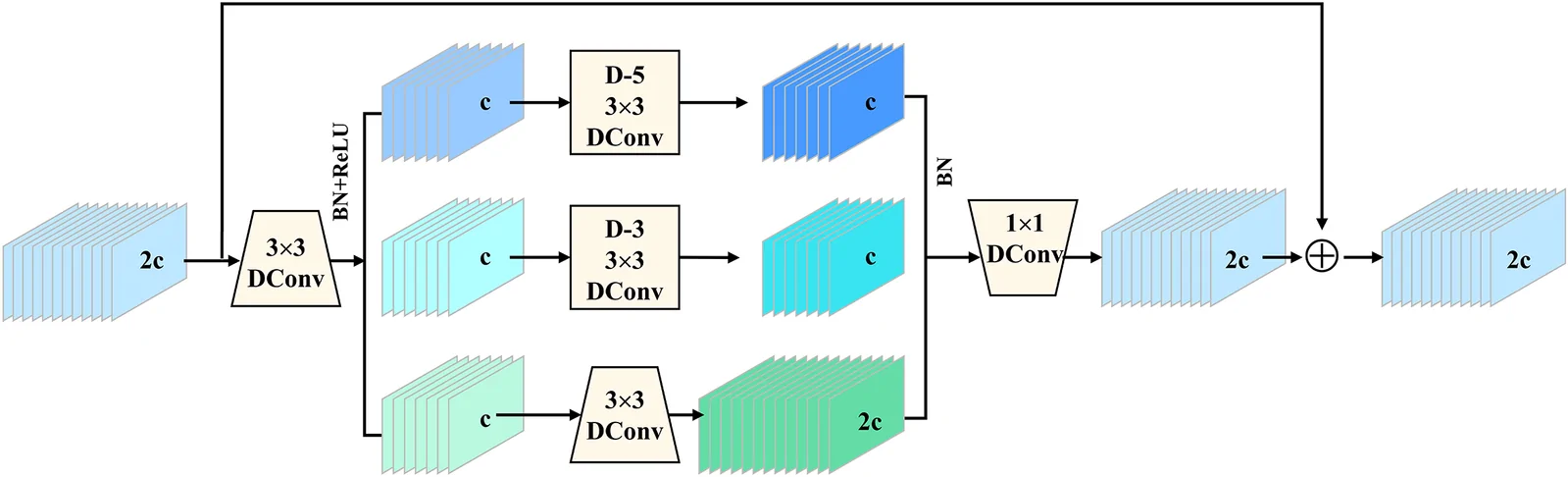
Structure of the dilation-wise residual module.

Let the input feature be $X\in {\mathbb{R}}^{C\times H\times W}$. A 3 × 3 convolution is first used to extract basic features and reduce the number of channels from *C* to *C*/2, as shown in Equation (1):

(1) $$F_{0}={\mathrm{Conv}}_{3\times 3}^{C\to C/2}\left(X\right)$$

The resulting feature *F*_0_ is then passed through three parallel convolutional branches with dilation rates of 1, 3, and 5, as shown in Equation (2):

(2) $$F_{r}={\mathrm{Conv}}_{3\times 3,r}^{C/2\to C_{r}}\left(F_{0}\right),r\in \left\{1,3,5\right\}$$

where *r* is the dilation rate. When *r* = 1, the number of output channels is *C*_r_ = *C*; when *r* = 3 or *r* = 5, *C*_r_ = *C*/2. The branch with *r* = 1 focuses on local textures and edge information, whereas the branches with *r* = 3 and *r* = 5 capture contextual information over larger receptive fields.

The outputs of the three branches are concatenated along the channel dimension, fused using a 1 × 1 convolution, and combined with the input through a residual connection, as shown in Equation (3):

(3) $$F_{\text{out}}=X+{\mathrm{Conv}}_{1\times 1}^{2C\to C}\left[\mathrm{Concat}\left(F_{1},F_{3},F_{5}\right)\right]$$

Here, Conv denotes a basic convolutional block comprising convolution, batch normalization, and an activation function. Four C3k2_DWR modules were deployed in the high-resolution P2 branch and at the bottom-up P3, P4, and P5 fusion nodes to extract local details and contextual information from fused features at different scales.

#### 2.2.4 SimAM attention mechanism

Early tobacco lesions, small insect-feeding holes, and local tears occupy relatively few pixels and are susceptible to interference from vein textures, complex backgrounds, and illumination variation. Therefore, SimAM attention was introduced after the C3k2_DWR modules in the P2 (stride 4) and P3 (stride 8) branches. SimAM is a three-dimensional attention mechanism that introduces no additional learnable parameters (Fan et al., 2023). It uses an energy function to measure the difference between a target neuron and the other neurons within the same channel. The minimum energy of the target neuron is defined in Equation (4):

(4) $$e_{t}^{*}=\frac{4\left({\hat{\sigma }}^{2}+\lambda \right)}{{\left(t\hat{\mu }\right)}^{2}+2{\hat{\sigma }}^{2}+2\lambda }$$

where *t* denotes the target neuron, $\hat{\mu }$ and ${\hat{\sigma }}^{2}$ are the mean and variance of the neurons in the same channel, respectively, and λ is a regularization parameter. The reciprocal of the minimum energy is mapped through a Sigmoid function to obtain the attention weights, yielding the enhanced feature as shown in Equation (5):

(5) $$X^{\prime }=\text{Sigmoid}\left(\frac{1}{E}\right)⊙X$$

where *X* and *X’* denote the input and weighted features, respectively; *E* is the tensor formed by the minimum energy values of all neurons; and ⊙ denotes element-wise multiplication. The SimAM weighting process is illustrated in **Figure 5**. This module adaptively weights high-resolution fused features to enhance the responses of lesions, insect-feeding holes, and mechanically damaged regions.

**Figure 5:**
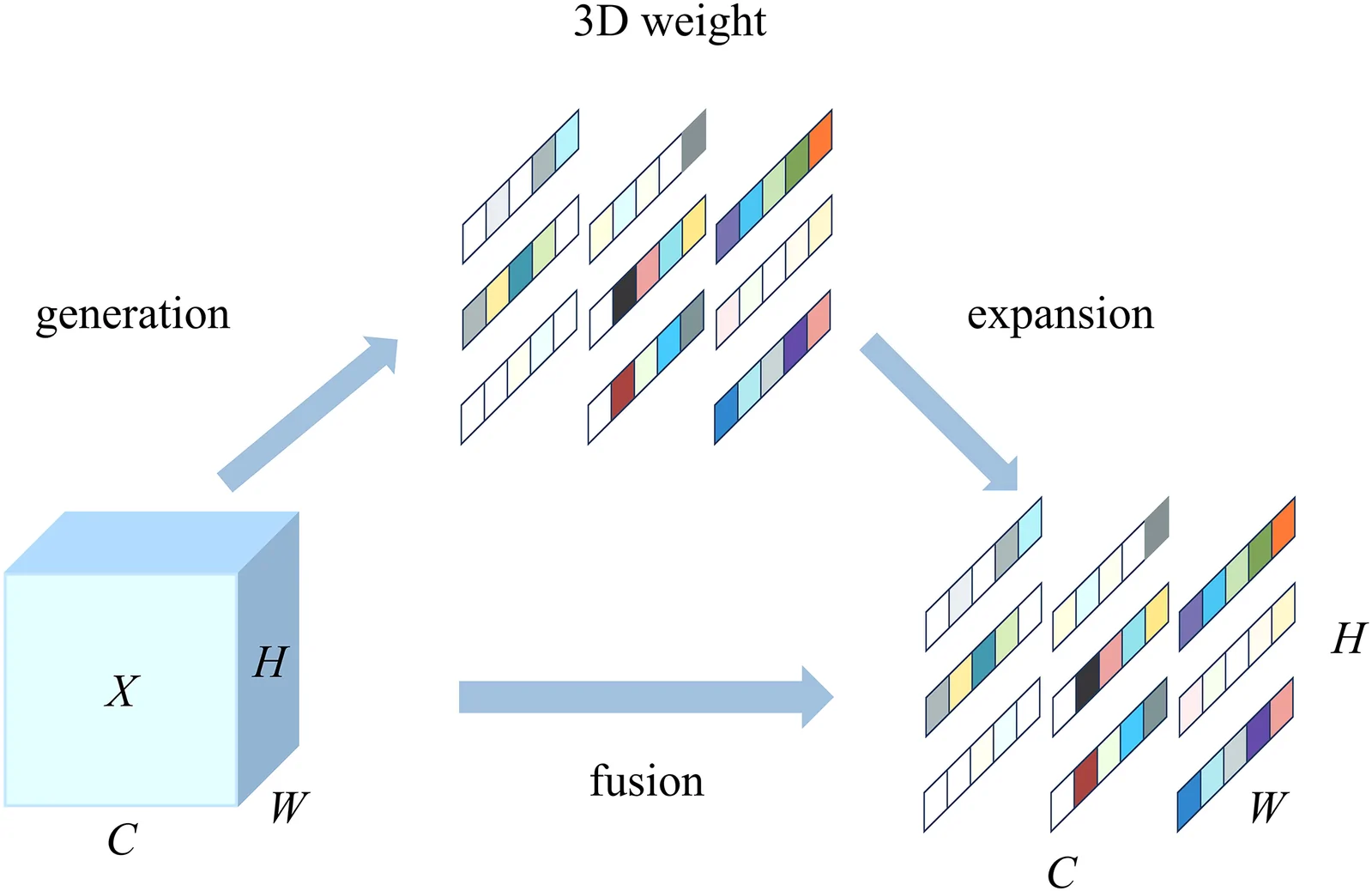
Schematic of three-dimensional attention weight generation in SimAM.

### 2.3 Secondary classification and quantification of mechanically damaged tobacco leaves

#### 2.3.1 Two-stage integration and reference-midrib extraction

The proposed method follows an integrated two-stage processing pipeline. In stage I, TLD-YOLO11n detects tobacco diseases, pests, and mechanical damage. When mechanical damage is detected, the corresponding whole-leaf image is passed to stage II; otherwise, no stage II analysis is performed. The mechanical damage decision threshold and joint evaluation procedure are described in the subsection “Mechanical damage screening and joint two-stage evaluation.” Because midrib continuity, leaf length, maximum width, and visible area depend on the complete leaf structure, stage II processes the whole-leaf image rather than a local crop extracted from the mechanical damage bounding box.

First, the image was converted to the hue–saturation–value (HSV) color space, and the leaf region was extracted using color thresholding. After morphological denoising, the largest connected component was retained as the leaf mask. Principal component analysis was then used to determine the longitudinal axis of the leaf. The image was rotated to normalize its orientation and resized to a standardized processing size while preserving its aspect ratio. A normalized midrib-response map was constructed by integrating leaf brightness, yellow–blue chromaticity, inverse saturation response, multiscale bright-ridge response, and longitudinal continuity. Responses near the leaf margin were suppressed to reduce interference from the leaf contour, lateral veins, and local textures.

Candidate points with high midrib responses were extracted from the central region of the leaf. A reference midrib was fitted using a weighted quadratic polynomial, and the fitting stability was improved through iterative outlier removal and neighborhood-based refinement. A narrow validation corridor was constructed along the reference midrib. Thirteen features describing midrib continuity and leaf morphology, including midrib response, dark-pixel proportion, transverse dark-band span, background-gap width, and leaf-occupancy ratio, were extracted and used as inputs to the mechanical damage classifier (Zhu et al., 2020; Xu et al., 2021).

#### 2.3.2 Midrib breakage classification and lamina loss quantification

Midrib breakage is primarily characterized by consecutive low responses, transverse dark bands, or background gaps within the reference-midrib corridor, or by the premature termination of the stable midrib segment connected to the petiole within the main leaf region. A binary logistic regression classifier was constructed using features describing midrib continuity, dark bands, background gaps, and leaf morphology. After feature standardization, the probability of midrib breakage was calculated using Equation (6):

(6) $$P_{\text{break}}={\left[1+\exp\left(-\beta_{0}-\sum_{i=1}^{n}\beta_{i}z_{i}\right)\right]}^{-1}$$

where *z_i_* is the standardized feature, and *β_i_* and *β*_0_ are the model coefficient and intercept, respectively. The classification threshold was selected using the validation set. An image was classified as midrib breakage when *P*_break_ was equal to or greater than the threshold; otherwise, it was classified as lamina loss.

Because midrib breakage disrupts the main supporting structure of the tobacco leaf, it was operationally categorized as severe mechanical damage. For leaves classified as lamina loss, the longitudinal leaf axis and its perpendicular direction were determined from the stable lower section of the midrib. Leaf length *L* and maximum width *W* were then measured, and the intact leaf area was estimated using Equation (7) (Zhang et al., 2023; Zheng et al., 2015:

(7) $$A_{est}=0.6345LW$$

The relative lamina loss index was calculated calculated using Equation (8):

(8) $$R_{loss}=max\left[0,\frac{A_{est}-A_{visible}}{A_{est}}\right]\times 100\%$$

where *A*_est_ is the estimated intact leaf area, *A*_visible_ is the visible area represented by the leaf mask, and *R*_loss_ is the relative lamina loss index. This index quantifies the proportion of missing tissue relative to the estimated intact leaf area, with larger values indicating greater tissue loss. For operational classification under the conditions of this study, 10% was used as the threshold between mild and severe lamina loss. Lamina loss was classified as mild when *R*_loss_<10% and severe when *R*_loss_≥10%. Midrib breakage was directly categorized as severe mechanical damage, and the lamina loss index was not used to determine its severity. The complete processing pipeline is shown in **Figure 6**.

**Figure 6:**
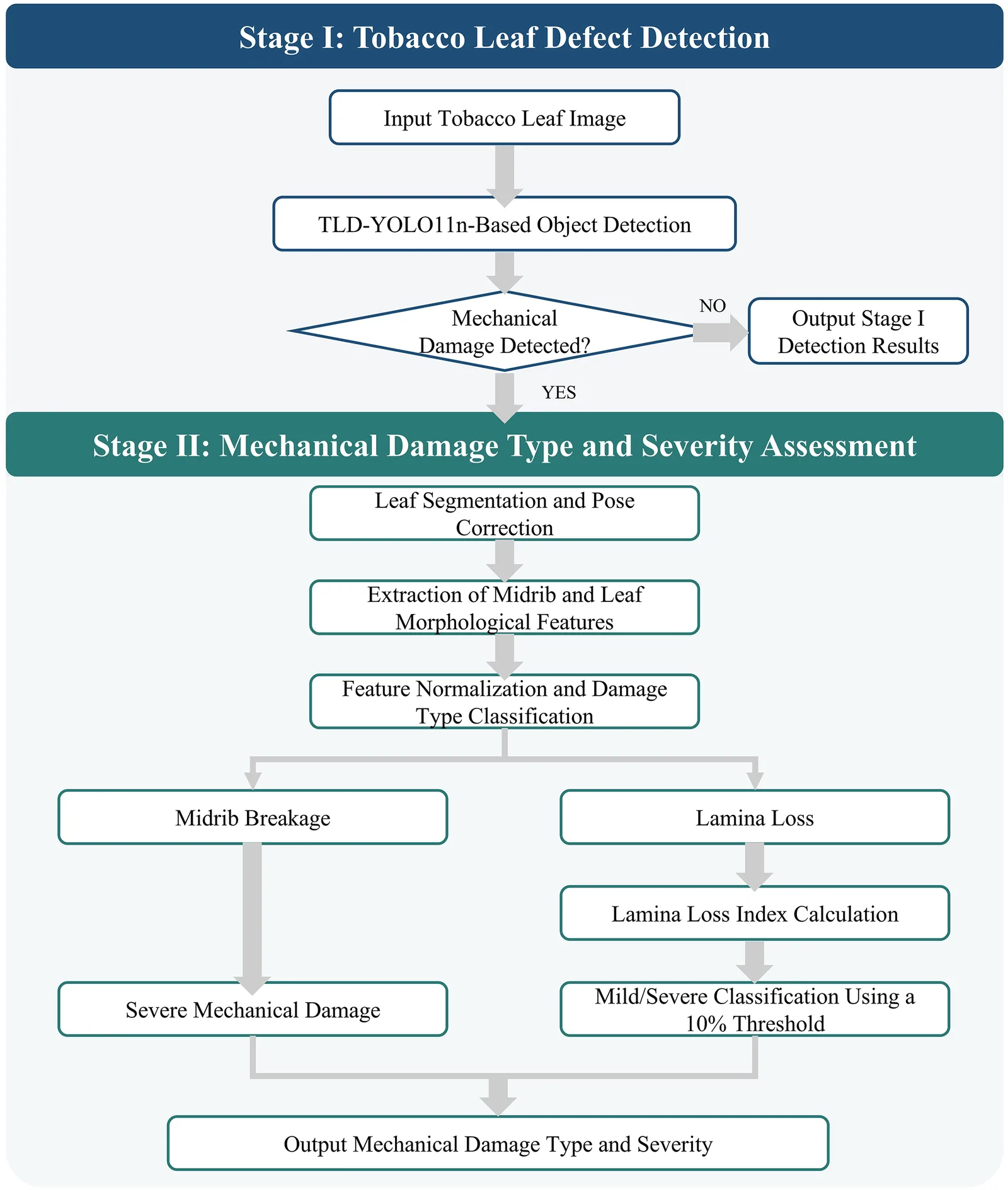
Two-stage pipeline for tobacco leaf defect detection and mechanical damage assessment.

### 2.4 Experimental environment and evaluation metrics

#### 2.4.1 Experimental environment

Model training and testing were conducted in a unified computing environment. The hardware platform comprised an Intel Core i9-13900HX processor and an NVIDIA GeForce RTX 4060 GPU. The software environment included Python 3.9, PyTorch 2.2.0, and the corresponding CUDA toolkit. To ensure comparability, all ablation and model-comparison experiments used the same hardware and software environment, dataset splits, and training parameters. The input image size was 640 × 640 pixels, the maximum number of training epochs was 300, the batch size was 16, the initial learning rate was 0.001, and Adam was used as the optimizer. Model-forward inference time was evaluated on the same computing platform with a batch size of 1 and FP32 precision. For the two-stage framework, the triggered processing time covered Stage I detection and routing, followed by Stage II feature extraction and damage-type classification.

#### 2.4.2 Evaluation metrics

Precision (*P*), recall (*R*), average precision (*AP*), and mean average precision (*mAP*) were used to evaluate detection performance. Model complexity and storage requirements were assessed using the number of parameters, model size, and giga floating-point operations (GFLOPs). Inference time (ms/image) was additionally used to evaluate computational efficiency. The metrics were calculated using Equations 9–12:

(9) $$Recall=\frac{TP}{TP+FN}\times 100\%$$

(10) $$Precision=\frac{TP}{TP+FP}\times 100\%$$

(11) $$AP=\int_{0}^{1}P\left(R\right)dR$$

(12) $$mAP=\frac{\sum_{i=1}^{K}AP_{i}}{K}\times 100\%$$

where *TP* is the number of true positives, *FP* is the number of false positives, *FN* is the number of false negatives, and *K* is the number of classes.

Additional statistical validation was performed for TLD-YOLO11n. Five-fold cross-validation was conducted using the combined training and validation data, and the results were reported as mean ± standard deviation (SD). Samples derived from the same original image were treated as one group throughout cross-validation and resampling analyses. On the test set, 95% confidence intervals (CIs) for the key detection metrics were estimated using 5000 bootstrap resamples. Differences between TLD-YOLO11n and YOLO11n in mAP_0.5_ and mAP_0.5:0.95_ were evaluated using two-sided paired permutation tests with 10000 Monte Carlo permutations. Holm adjustment was applied to the two prespecified comparisons, with adjusted p< 0.05 considered statistically significant.

#### 2.4.3 Mechanical damage screening and joint two-stage evaluation

To evaluate stage I mechanical damage screening and the coordination between the two stages, image-level screening was performed on all 206 test images. The 50 mechanical damage images were treated as positive samples, and the remaining 156 disease and pest images were treated as negative samples. An image was forwarded to stage II when it contained at least one predicted mechanical damage bounding box with a confidence score of 0.25 or higher. This threshold was fixed before testing, and the test set was not used for threshold optimization. True positives, false positives, true negatives, and false negatives were determined from the ground-truth mechanical damage labels and whether each image was forwarded to stage II. Precision and recall were calculated as defined above, while the F1-score, accuracy, and specificity were calculated using Equations 13–15:

(13) $$F1\text{-score}=\frac{2PR}{P+R}$$

(14) $$\text{Accuracy}=\frac{N_{\text{correct}}}{N}\times 100\%$$

(15) $$\text{Specificity}=\frac{TN}{TN+FP}\times 100\%$$

Here, *P* and *R* denote the precision and recall of mechanical damage screening, respectively; *TN* is the number of disease and pest images that were not forwarded to stage II; *N*_correct_ is the number of correctly screened images; and *N* is the total number of images in the full test set. All values were reported as percentages.

Stage II damage-type classification was evaluated using ground-truth mechanical damage images that were successfully processed in stage II. Accuracy and the class-specific precision, recall, and F1-score for lamina loss and midrib breakage were reported. A joint evaluation was further conducted on all 50 mechanical damage test images. A stage I miss, stage II processing failure, or incorrect damage-type classification was counted as a joint error. A result was considered jointly correct only when mechanical damage was correctly detected in stage I and the stage II output matched the image-level ground-truth label. Joint damage-type accuracy was defined as the proportion of jointly correct images among all mechanical damage test images.

## 3 Results and discussion

### 3.1 Ablation experiments on different modules

To evaluate the effects of the P2 detection head, CARAFE upsampling, C3k2_DWR feature extraction module, and SimAM attention mechanism on model performance, single-module and combined ablation experiments were conducted using YOLO11n as the baseline. All experiments used the same dataset split, training settings, and evaluation protocol. The results are presented in **Table 1**.

The single-module experiments showed that the four modifications affected model performance differently. Adding the P2 detection head increased recall, mAP_0.5_, and mAP_0.5:0.95_ by 1.3, 1.8, and 1.1 percentage points, respectively, indicating that the high-resolution detection branch facilitated the detection of small targets. However, GFLOPs increased from 6.4 to 10.4. C3k2_DWR achieved the highest values for both mAP metrics among the single-module configurations, improving mAP_0.5_ and mAP_0.5:0.95_ by 2.2 and 1.7 percentage points over the baseline, respectively, while increasing the parameter count and GFLOPs by only 0.033 M and 0.2. SimAM increased recall by 3.7 percentage points but reduced precision by 2.8 percentage points. The standalone introduction of CARAFE did not yield consistent improvements, suggesting that its effectiveness also depended on the feature hierarchy and subsequent fusion architecture.

Performance did not increase monotonically as more modules were added. Combining P2 with CARAFE increased precision to 84.8%, but recall and both mAP metrics were lower than those obtained with P2 alone. Adding C3k2_DWR partially recovered recall and both mAP metrics. When SimAM was added to the P2, CARAFE, and C3k2_DWR configuration, precision, recall, mAP_0.5_, and mAP_0.5:0.95_ reached 87.0%, 77.8%, 84.5%, and 64.1%, respectively, representing gains of 4.3, 1.8, 2.1, and 2.6 percentage points over the corresponding configuration without SimAM. Thus, SimAM simultaneously improved classification precision, target recall, and bounding-box localization under this combined configuration.

Compared with YOLO11n, the final model improved precision, recall, mAP0.5, and mAP0.5:0.95 by 3.5, 4.1, 4.0, and 3.9 percentage points, respectively. The parameter count and model size increased by 0.256 M and 0.6 MB, respectively, while GFLOPs increased by 5.1. The increase in computational cost was mainly associated with the addition of the high-resolution P2 branch, whereas the performance of the combined model was consistent with configuration-dependent and non-additive effects among the modified feature extraction and fusion components, rather than a simple accumulation of independent module gains. Considering detection performance, parameter count, and storage requirements, the combination of P2, CARAFE, C3k2_DWR, and SimAM was selected as the final TLD-YOLO11n architecture.

### 3.2 Comparative experiments on key modules

#### 3.2.1 Comparison of different upsampling methods

To determine the specific configurations of the upsampling method, C3k2 feature extraction module, and attention mechanism in TLD-YOLO11n, alternative modules of the same type were compared while the remaining network architecture and training conditions were held constant. For the upsampling comparison, the P2 detection head, C3k2_DWR, and SimAM were fixed, and nearest-neighbor interpolation, DySample (Liu et al., 2023), and CARAFE were evaluated. The results are shown in **Table 2**.

Compared with nearest-neighbor interpolation, DySample improved precision, mAP_0.5_, and mAP_0.5:0.95_ by 3.4, 2.1, and 1.2 percentage points, respectively, while leaving the parameter count, GFLOPs, and model size nearly unchanged. Recall increased by only 0.1 percentage points. CARAFE achieved the same precision as DySample but further improved recall, mAP_0.5_, and mAP_0.5:0.95_ by 2.5, 1.1, and 1.6 percentage points, respectively. These results suggest that the main benefits of CARAFE were improved target detection and more accurate bounding-box localization at stricter intersection-over-union thresholds. Compared with nearest-neighbor interpolation, CARAFE added 0.144 M parameters, 0.8 GFLOPs, and 0.2 MB of model size, while increasing mAP_0.5_ and mAP_0.5:0.95_ by 3.2 and 2.8 percentage points, respectively. Based on the balance between detection performance and model complexity, CARAFE was selected for feature upsampling in the top-down pathway of the TLD-YOLO11n neck.

#### 3.2.2 Comparison of different C3k2 feature extraction modules

With the P2 detection head, CARAFE upsampling, and SimAM attention fixed, the original C3k2, C3k2_FDConv (Chen et al., 2025), C3k2_RepViTBlock (Wang et al., 2024a), C3k2_FourierSR (Li et al., 2026), and C3k2_DWR were compared. The results are presented in **Table 3**.

The C3k2 variants exhibited different trade-offs among precision, recall, localization performance, and model complexity. C3k2_FDConv achieved the highest recall at 79.6%, but its precision was 82.1% and its model size increased to 7.8 MB, indicating a trade-off between higher recall and lower precision. C3k2_RepViTBlock and C3k2_FourierSR both reduced the parameter count and GFLOPs, but neither surpassed the original C3k2 in either mAP metric. In particular, the mAP_0.5:0.95_ of C3k2_FourierSR decreased to 59.5%. C3k2_DWR achieved the highest precision, mAP_0.5_, and mAP_0.5:0.95_, reaching 87.0%, 84.5%, and 64.1%, respectively. Its recall was 77.8%, only 1.8 percentage points lower than that of C3k2_FDConv. Compared with the original C3k2, C3k2_DWR improved precision, recall, mAP_0.5_, and mAP_0.5:0.95_ by 2.0, 3.5, 2.4, and 3.5 percentage points, respectively, while adding only 0.035 M parameters and 0.3 GFLOPs. Compared with C3k2_FDConv, C3k2_DWR reduced the model size by 1.9 MB and improved precision, mAP_0.5_, and mAP_0.5:0.95_ by 4.9, 1.2, and 2.6 percentage points, respectively. Considering both detection performance and model complexity, C3k2_DWR was selected as the feature extraction module for subsequent experiments.

#### 3.2.3 Comparison of different attention mechanisms

To identify a suitable attention mechanism for the improved model, Convolutional Block Attention Module (CBAM) (Woo et al., 2018), Normalization-based Attention Module (NAM) (Liu et al., 2021), Efficient Multi-Scale Attention (EMA) (Ouyang et al., 2023), and SimAM were separately introduced into the P2–CARAFE–C3k2_DWR configuration. The dataset split, training parameters, and module insertion positions were kept identical across experiments. The results are presented in **Table 4**.

The attention mechanisms affected precision and recall differently. Compared with the configuration without attention, CBAM slightly improved all four detection metrics, including a 0.8-percentage-point increase in mAP_0.5:0.95_. NAM increased recall and mAP_0.5_ by 1.1 and 0.9 percentage points, respectively, but reduced precision by 1.0 percentage points. EMA improved precision, mAP_0.5_, and mAP_0.5:0.95_ by 3.8, 1.7, and 1.3 percentage points, respectively, while reducing recall by 0.8 percentage points. SimAM achieved the highest values for all four detection metrics, with precision, recall, mAP_0.5_, and mAP_0.5:0.95_ of 87.0%, 77.8%, 84.5%, and 64.1%, respectively. These values were 4.3, 1.8, 2.1, and 2.6 percentage points higher than those obtained without attention. At the reported numerical precision, SimAM did not increase the parameter count, GFLOPs, or model size. Its mean model-forward inference time was 6.3 ms/image, compared with 6.0 ms/image for the configuration without attention, indicating an additional latency of approximately 0.3 ms/image. Considering its improvements in all four detection metrics together with this limited additional inference latency, SimAM was adopted to reweight the high-resolution fused features at P2 and P3 in TLD-YOLO11n.

### 3.3 Comparison of different YOLO models

To evaluate the overall detection performance of TLD-YOLO11n, YOLOv5n, YOLOv6n, YOLOv8n, YOLOv9t, YOLOv10n, YOLO11n, YOLOv12n, YOLO26n, and the recently proposed TGL-YOLO were selected as comparison models (Wang and Wang, 2026). For TGL-YOLO, the nano-scale (n) configuration was adopted to maintain a comparable lightweight model scale. All models were trained and tested using the same dataset split, input image size, number of training epochs, major training parameters, experimental environment, and evaluation criteria. The results are presented in **Table 5**.

The models exhibited different trade-offs between detection performance and complexity. Among the comparison models other than TLD-YOLO11n, YOLOv9t achieved the highest recall, mAP_0.5_, and mAP_0.5:0.95_, at 76.5%, 81.0%, and 61.0%, respectively. YOLO26n had one of the lowest computational costs, with 5.8 GFLOPs, but its mAP_0.5:0.95_ was only 58.6%. TGL-YOLO achieved a precision of 81.1%, a recall of 74.2%, an mAP_0.5_ of 78.4%, and an mAP_0.5:0.95_ of 59.0%, with 2.618 M parameters and 5.8 GFLOPs. TLD-YOLO11n achieved the highest precision, recall, mAP_0.5_, and mAP_0.5:0.95_ among all models, reaching 87.0%, 77.8%, 84.5%, and 64.1%, respectively. Compared with the original YOLO11n, these metrics increased by 3.5, 4.1, 4.0, and 3.9 percentage points, respectively. Compared with TGL-YOLO, TLD-YOLO11n improved mAP_0.5_ and mAP_0.5:0.95_ by 6.1 and 5.1 percentage points, respectively. Relative to the best mAP results among the other models, TLD-YOLO11n improved mAP_0.5_ and mAP_0.5:0.95_ by 3.5 and 3.1 percentage points, respectively. TLD-YOLO11n contained 2.847 M parameters, required 11.5 GFLOPs, and had a model size of 5.9 MB. Its mean model-forward inference time was 6.3 ms/image, compared with 5.3 ms/image for the original YOLO11n. Its parameter count and storage requirement were comparable to those of most lightweight YOLO models, although its computational cost was higher. Overall, TLD-YOLO11n achieved improved multiclass tobacco leaf defect detection and localization performance at the cost of a moderate increase in computation.

In the five-fold cross-validation, TLD-YOLO11n achieved a precision of 85.8 ± 1.0%, a recall of 78.3 ± 1.0%, an mAP_0.5_ of 85.6 ± 1.3%, and an mAP_0.5:0.95_ of 65.7 ± 1.4% (mean ± SD). On the test set, the mAP_0.5_ values of YOLO11n and TLD-YOLO11n were 80.5% (95% CI: 75.8–85.8%) and 84.5% (95% CI: 81.5–88.7%), respectively; the corresponding mAP_0.5:0.95_ values were 60.2% (95% CI: 56.3–65.1%) and 64.1% (95% CI: 60.5–68.9%). The paired improvements were 4.0 percentage points for mAP_0.5_ (95% CI: 1.6–6.9 percentage points; Holm-adjusted p = 0.0102) and 3.9 percentage points for mAP_0.5:0.95_ (95% CI: 1.9–6.0 percentage points; Holm-adjusted p = 0.0018).

To examine class-specific differences before and after model improvement, normalized confusion matrices were generated for YOLO11n and TLD-YOLO11n, as shown in **Figure 7**. The diagonal proportion for each of the five classes was higher for TLD-YOLO11n than for YOLO11n. The proportions for oriental tobacco budworm and tobacco wildfire disease increased by 7 and 6 percentage points, respectively. The proportion of each ground-truth class assigned to the background decreased by 3–7 percentage points, consistent with the overall improvement in recall. Interclass confusion was limited for both models. However, background regions were still relatively frequently misclassified as oriental tobacco budworm, suggesting that some leaf textures, partially occluded regions, or small background structures produced feature responses similar to those of the pest targets.

**Figure 7:**
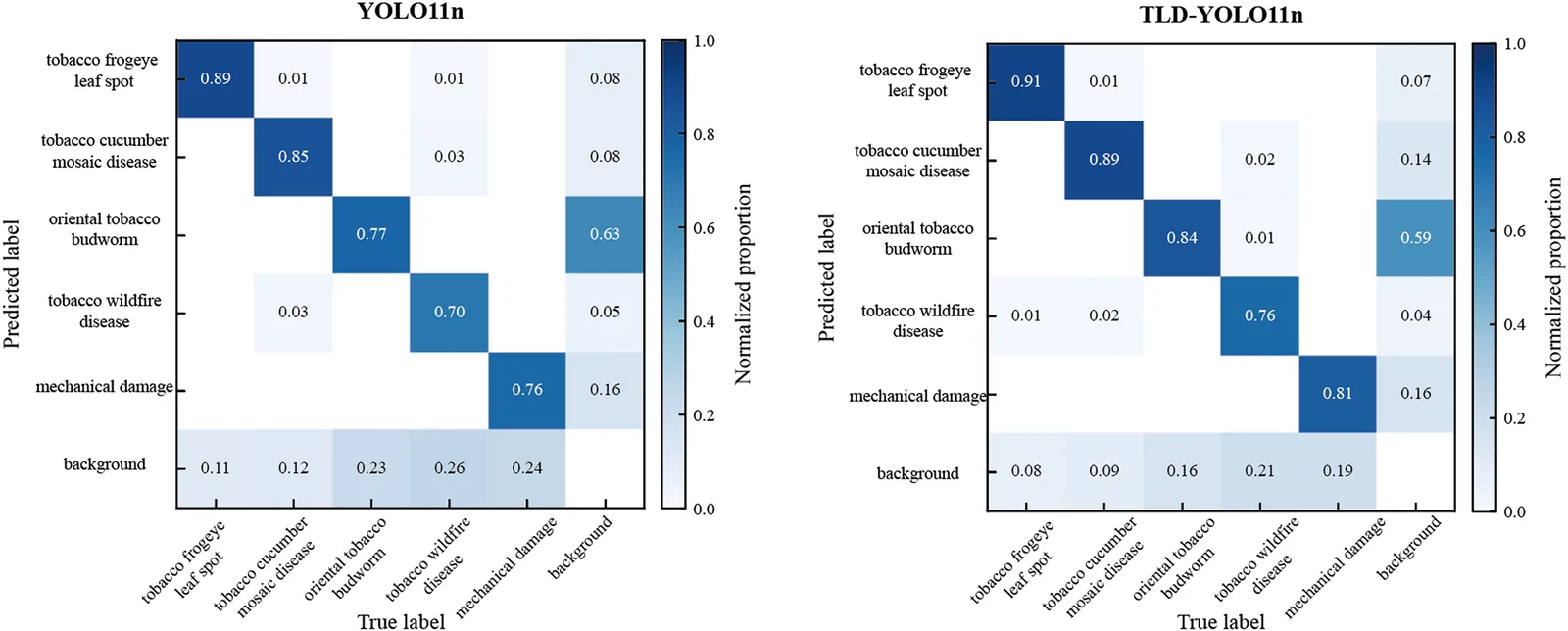
Normalized confusion matrices of YOLO11n and TLD-YOLO11n.

**Figure 8** presents detection results for representative test images from the five classes. Both models detected the major targets in the tobacco frogeye leaf spot, tobacco cucumber mosaic disease, tobacco wildfire disease, and mechanical damage images, although some bounding boxes produced by TLD-YOLO11n had higher confidence scores. In the image containing densely distributed oriental tobacco budworms, TLD-YOLO11n detected more targets than YOLO11n. These visual results are consistent with the increased recall reported in **Table 5** and the reduced proportion of missed detections shown in **Figure 7**.

**Figure 8:**
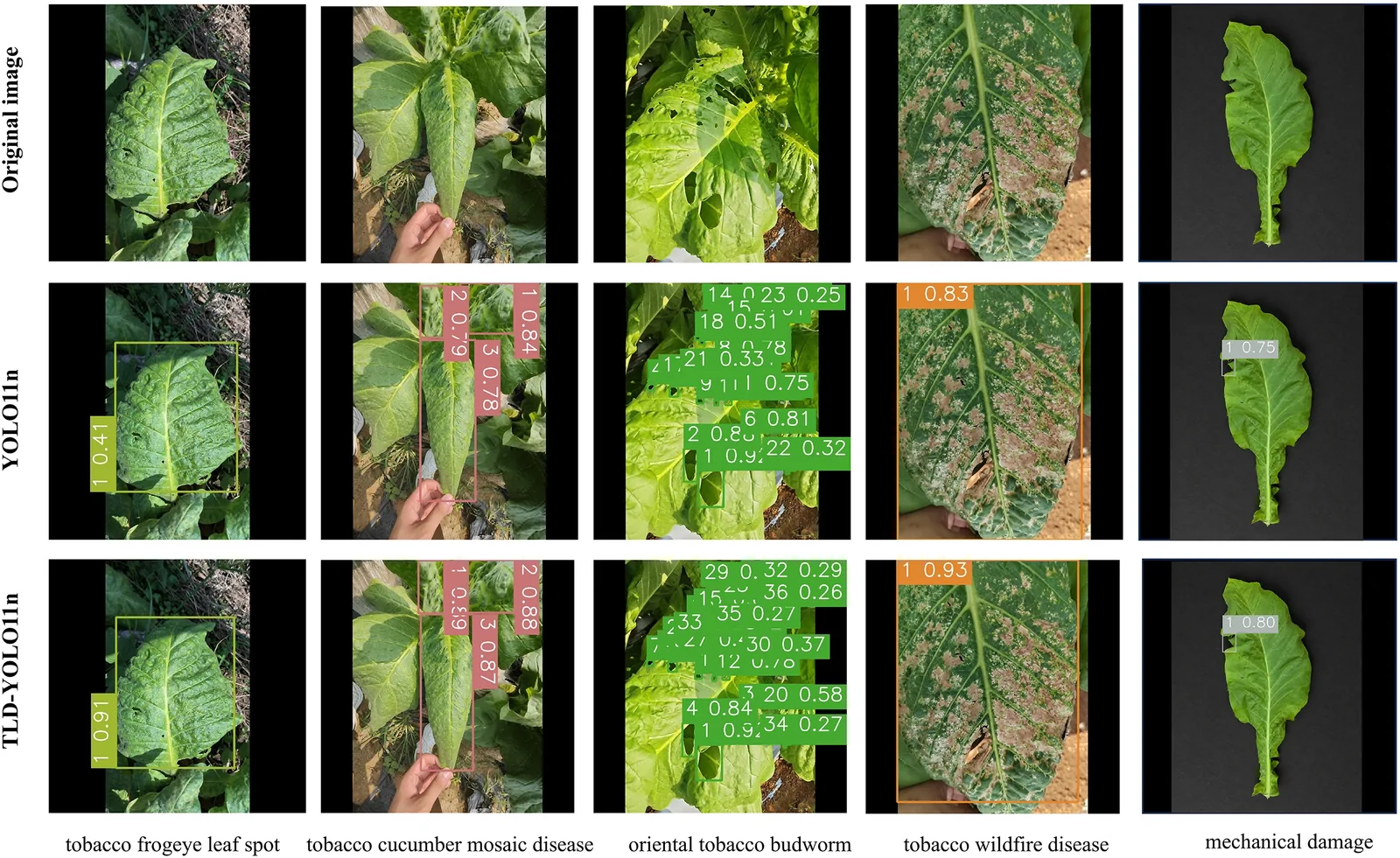
Representative detection results of YOLO11n and TLD-YOLO11n for different tobacco leaf defect classes.

### 3.4 Two-stage cascade evaluation and lamina loss quantification

Following the evaluation procedure described in the Methods subsection “Mechanical damage screening and joint two-stage evaluation,” the stage II logistic regression classifier was trained and its parameters were selected. The initial classifier was fitted using 350 training images, while 100 validation images were used to select the regularization parameter, class-weighting strategy, and classification threshold. The 50 test images were not used to calculate feature-standardization parameters, fit the classifier, or select the threshold. Validation-set evaluation resulted in a regularization parameter of *C* = 100, balanced class weights, and a midrib breakage probability threshold of 0.44. After these settings had been determined, the training and validation sets were combined, the feature-standardization parameters were recalculated, and the final classifier was fitted.

To evaluate stage I mechanical damage screening and the coordination between the two stages, image-level screening was conducted on the full test set of 206 images. The 50 images showing mechanical damage were treated as positive samples, comprising 33 midrib breakage images and 17 lamina loss images, while the remaining 156 disease- and pest-affected images were treated as negative samples. At the fixed mechanical damage confidence threshold of 0.25, all 50 positive images were correctly identified and passed to stage II, whereas none of the 156 negative images were passed to the second stage. The resulting numbers of true positives, false positives, true negatives, and false negatives were 50, 0, 156, and 0, respectively. Accordingly, the precision, recall, specificity, and F1-score of stage I mechanical damage screening were all 100.0%. Thus, no mechanical damage image was missed, and no disease- or pest-affected image was incorrectly passed to stage II within the current combined test set. The screening and damage-type classification results are summarized in **Table 7**.

**Table 7:** Performance of mechanical damage screening and stage II damage-type classification.

Evaluation level	Task or class	P (%)	R (%)	Specificity (%)	F1-score (%)	Number of images
Stage I screening	Mechanical damage screening	100.0	100.0	100.0	100.0	206
Stage II classification	Lamina loss	88.9	94.1	–	91.4	17
	Midrib breakage	96.9	93.9	–	95.4	33

All 50 mechanical damage test images were successfully processed in stage II, with no stage I misses or stage II processing failures. Of the 17 lamina loss images, 16 were correctly classified and one was misclassified as midrib breakage. Of the 33 midrib breakage images, 31 were correctly classified and two were misclassified as lamina loss. The stage II damage-type classification accuracy was 94.0%. Recall was 94.1% for lamina loss and 93.9% for midrib breakage, while the corresponding F1-scores were 91.4% and 95.4%. The lower precision of the lamina loss class was primarily attributable to the two midrib breakage images that were misclassified as lamina loss.

In the joint two-stage evaluation of the mechanical damage test subset, no Stage I misses or stage II processing failures occurred, and the damage types of 47 of the 50 images were correctly classified. The resulting joint damage-type classification accuracy was therefore 94.0%, identical to the conditional stage II classification accuracy. All three joint errors originated from stage II misclassification: two midrib breakage images were classified as lamina loss, and one lamina loss image was classified as midrib breakage. Qualitative examination of these misclassified samples showed that the two damage types could exhibit overlapping structural characteristics. In the two midrib breakage cases classified as lamina loss, the damage was accompanied by conspicuous transverse or marginal lamina tearing, while the remaining midrib segments retained relatively good spatial alignment, making the overall morphology resemble lamina loss. Conversely, in the lamina loss case classified as midrib breakage, the missing-tissue region was located close to the midrib and formed a local gap resembling an interruption of midrib continuity. These observations indicate that the remaining Stage II errors were concentrated in cases where the structural manifestations of midrib breakage and lamina loss overlapped. The computational performance of the two-stage framework was also evaluated. For mechanically damaged images routed from Stage I to Stage II, the mean processing time from Stage I detection and routing through Stage II feature extraction and damage-type classification was 152.4 ms/image.

**Figure 9** illustrates the two-stage processing results for three representative damage conditions. From left to right, each row shows the original tobacco leaf image, the TLD-YOLO11n mechanical damage detection result, the normalized midrib-response map, and the damage-type and severity classification result. After mechanical damage was detected in stage I, the whole-leaf image was passed to stage II. Midrib breakage was characterized by an evident interruption or premature termination of the response near the reference midrib. In contrast, the reference midrib generally remained continuous in lamina loss samples, in which the damage was mainly manifested as missing tissue along the leaf margin or within the lamina.

**Figure 9:**
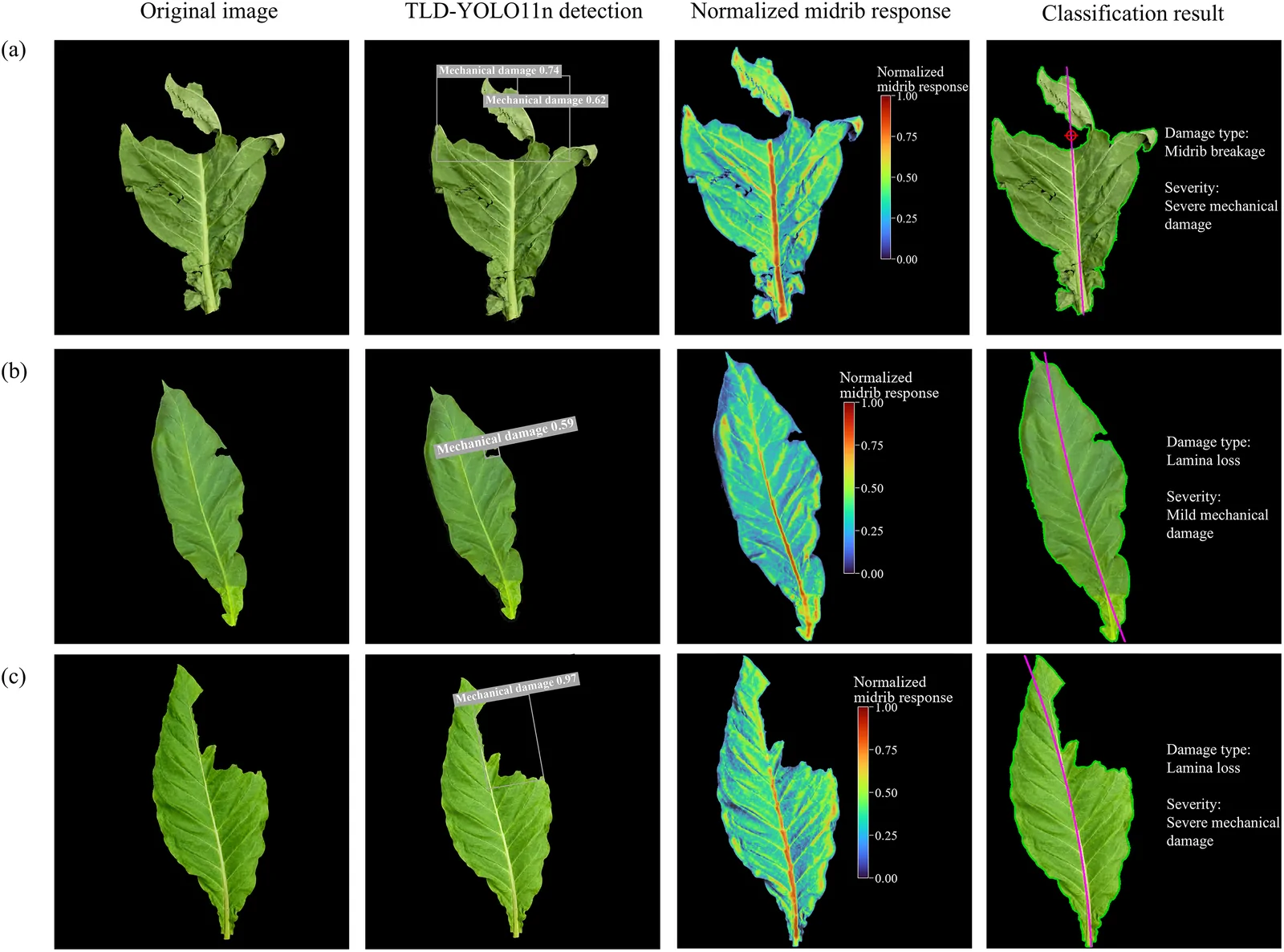
Representative results of two-stage mechanical damage detection and assessment: **(a)** midrib breakage; **(b)** mild lamina loss; and **(c)** severe lamina loss.

According to the operational classification rules described in the Methods subsection “Midrib breakage classification and lamina loss quantification,” leaves with midrib breakage were categorized as having severe mechanical damage. For leaves classified as lamina loss, leaf length, maximum width, and the relative lamina loss index were calculated with reference to the stable midrib direction. The relative lamina loss index of the leaf in **Figure 10A** was 4.42%, which was below the operational threshold of 10%; the leaf was therefore classified as exhibiting mild lamina loss. The index for the leaf in **Figure 10B** was 20.21%, resulting in its classification as severe lamina loss. These examples illustrate that the index responds differently to different levels of lamina tissue loss and provides a quantitative basis for the operational classification of lamina-loss severity.

**Figure 10:**
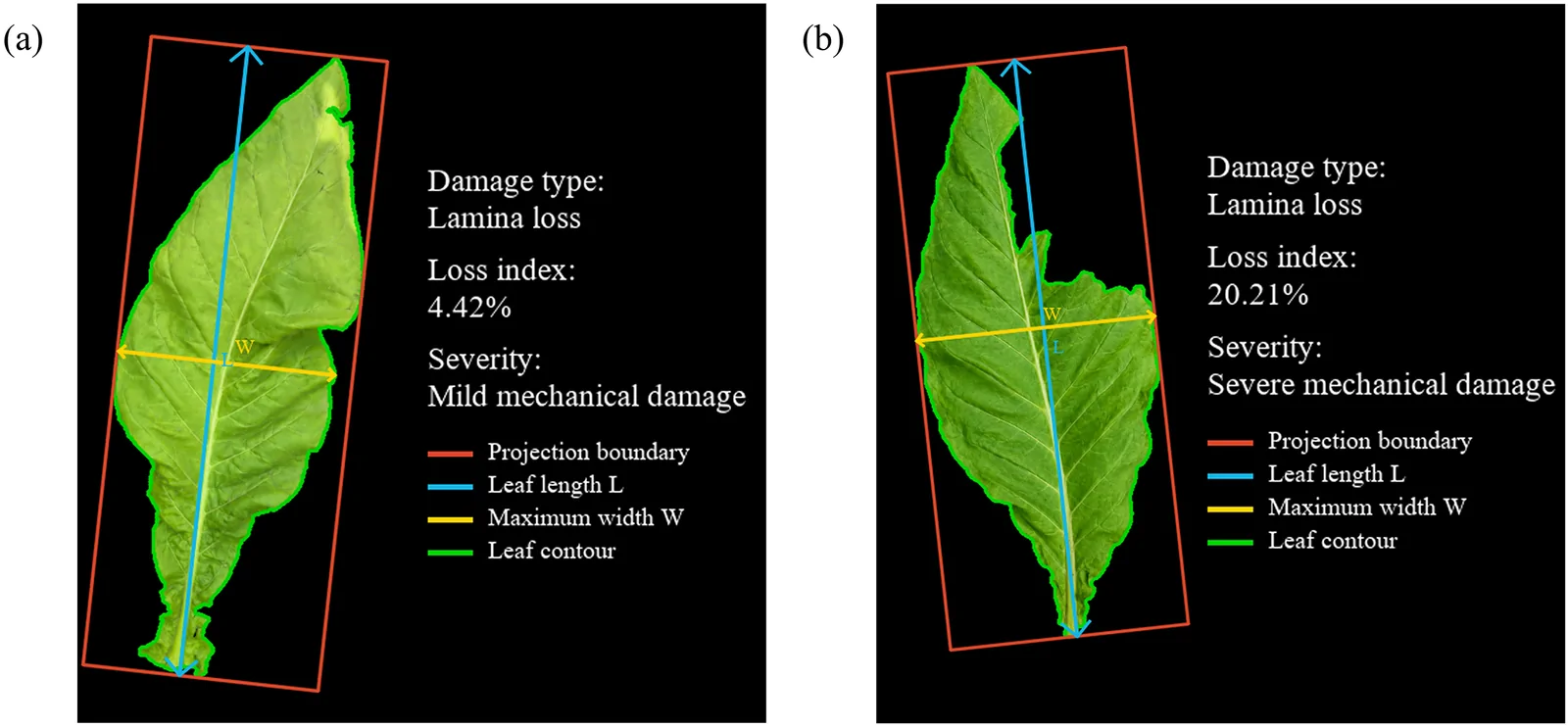
Lamina loss quantification based on leaf-length and leaf-width projections aligned with the stable midrib direction: **(a)** mild lamina loss; and **(b)** severe lamina loss.

Recent studies in tobacco machine vision and quantitative leaf-damage assessment have increasingly extended visual analysis from categorical recognition toward harvesting-quality monitoring and damage quantification. Object detection combined with digital-twin technology has been applied to identify operational abnormalities during tobacco harvesting (You et al., 2026), while an improved YOLOv5 detector combined with multi-frame tracking has been used to estimate the overall damage rate of fresh tobacco leaves in mechanical-harvesting scenarios (Yu et al., 2026). In addition, deep learning and image processing have been applied to quantitatively estimate herbivory-induced leaf damage (Wang et al., 2024b). These studies indicate that visual assessment is increasingly moving beyond defect recognition alone toward more quantitative quality evaluation. The present two-stage framework addresses a complementary aspect of this problem by first screening multiple tobacco-leaf defect types and then distinguishing midrib breakage from lamina loss at the individual-leaf level, while providing a relative quantitative measure of lamina loss.

## 4 Conclusions

This study developed a tobacco leaf defect detection and mechanical damage assessment method that combines object detection with secondary damage classification. In stage I, TLD-YOLO11n detects tobacco frogeye leaf spot, tobacco cucumber mosaic disease, oriental tobacco budworm, tobacco wildfire disease, and mechanical damage. When mechanical damage is detected, the corresponding whole-leaf image is passed to stage II. Reference-midrib and leaf-morphology features are then used to distinguish midrib breakage from lamina loss and to provide a relative measure of lamina loss and support its operational severity classification. The method may provide a reference for defect screening and structural damage assessment of mechanically harvested tobacco leaves. The main conclusions are as follows:

1. Following the introduction of a P2 detection head, CARAFE upsampling, C3k2_DWR feature extraction, and SimAM attention into YOLO11n, TLD-YOLO11n achieved precision, recall, mAP_0.5_, and mAP_0.5:0.95_ values of 87.0%, 77.8%, 84.5%, and 64.1%, respectively. These values exceeded those of the original YOLO11n by 3.5, 4.1, 4.0, and 3.9 percentage points, respectively. The ablation and alternative-module comparisons showed that the selected architecture improved the overall detection of tobacco leaf defects with different scales and morphologies.
2. Among the YOLO models evaluated, TLD-YOLO11n achieved the highest precision, recall, mAP_0.5_, and mAP_0.5:0.95_. Its two mAP values exceeded the best results of the other models by 3.5 and 3.1 percentage points, respectively. The confusion matrices and representative detection results indicated that the improvements reduced the proportion of targets missed as background and enhanced the detection of densely distributed oriental tobacco budworms.
3. On the full test set, the precision, recall, specificity, and F1-score of stage I mechanical damage screening were all 100.0%, with no mechanical damage images missed and no disease- or pest-affected images incorrectly passed to stage II. All 50 images showing mechanical damage were processed in stage II, and 47 were assigned the correct damage type, resulting in a joint damage-type classification accuracy of 94.0%. The F1-scores for lamina loss and midrib breakage were 91.4% and 95.4%, respectively. For leaves classified as lamina loss, the relative lamina loss index provided a relative measure of tissue loss and supported operational classification into mild and severe categories using the predefined threshold.

The stage II mechanical damage analysis was evaluated primarily under controlled imaging conditions involving a black background and approximately flattened single leaves. In addition, the disease and pest images and the mechanical damage images originated from different sources and imaging conditions. Future work will therefore expand the dataset to include tobacco leaves from different production regions and cultivars and incorporate greater variation in leaf orientation, illumination, background, and imaging environments to evaluate the stability of the method under more complex practical conditions. In particular, Stage I defect detection will be further evaluated on tobacco plants under field and natural-background conditions, whereas the complete two-stage framework will be assessed using mechanically harvested leaves in continuous conveying and sorting scenarios, with particular attention to detection stability, computational efficiency, and real-time operational performance.

## Data Availability

The datasets presented in this study can be found in online repositories. The names of the repository/repositories and accession number(s) can be found below: https://github.com/Tobaccodefect/tobacco-leaf-defect-detection-and-damage-assessment.
